# Multivariate Statistical Analyses of the Temporal Variation in the Chemical Composition of the Essential Oil of *Eucalyptus torquata* in Cyprus

**DOI:** 10.3390/molecules30020332

**Published:** 2025-01-15

**Authors:** Mustapha Bulama Modu, Duygu Yiğit Hanoğlu, Azmi Hanoğlu, Fehmi Burak Alkaş, K. Hüsnü Can Başer, Dudu Özkum Yavuz

**Affiliations:** 1 Department of Pharmaceutical Botany, Faculty of Pharmacy, Near East University, 99010 Nicosia, Cyprus; mustapha.bulamamodu@neu.edu.tr (M.B.M.); dudu.ozkum@neu.edu.tr (D.Ö.Y.); 2 Department of Pharmacognosy, Faculty of Pharmacy, Near East University, 99010 Nicosia, Cyprus; azmi.hanoglu@neu.edu.tr (A.H.); kemalhusnucan.baser@neu.edu.tr (K.H.C.B.); 3 Department of Toxicology, Faculty of Pharmacy, Near East University, 99010 Nicosia, Cyprus; fehmibalkas@gmail.com

**Keywords:** *Eucalyptus torquata*, Cyprus, essential oil, principal component analysis, PCA, hierarchical cluster analysis, HCA, 1,8-cineole, torquatone

## Abstract

The genus *Eucalyptus* L’Hér., is native to Australia with 61 introduced taxa in Cyprus, including *E. torquata* Luehm., which has a wide distribution on the island. The aim of this study was to investigate the possible seasonal variations in the chemical composition of the essential oils of juvenile and mature leaves collected from Nicosia, Cyprus, by using multivariate statistical analysis. The leaves of 12 monthly collections were separately hydrodistilled, and GC-FID and GC/MS analyses were conducted. In general, the results revealed 1,8-cineole (mature: 3.6–27.8%; juvenile: 12.7–21.5%) and torquatone (mature: 27.6–48.8%; juvenile: 28.8–41.5%) as major compounds as well as an inverse relation between 1,8-cineole and torquatone content. Other important compounds found were α-pinene, β-eudesmol and α-eudesmol for all samples. The data support the existence of three major clusters, distinguished by the concentration of torquatone and miniatone. Minor compounds were also temporally relevant. The present study is among the first of its kind, analyzing the essential oils for a one-year period in Cyprus as well as conducting statistical analysis on *E. torquata* to reveal possible temporal variations between heterophyllous leaves, and also performing Hierarchical Cluster Analysis, determining the primary components of variability.

## 1. Introduction

The genus *Eucalyptus* L’Hér. is native to Australia and belongs to the Myrtaceae family, which comprises over 140 genera and approximately 3800 species, predominantly trees and shrubs characterized by oil glands in their leaves [1]. The medicinally important genus *Eucalyptus* comprises species that are ecologically significant. About 74% of Australian forests and woodlands are covered by *Eucalyptus* taxa [2]. Eucalypts (*Eucalyptus* spp.) are widely cultivated globally, particularly in the Mediterranean and subtropical regions [3]. The genus was introduced in various parts of the world in the late 19th century, primarily for its fast growth and utility in industries such as paper manufacturing and for its commercial value, with less than 20 species being exploited for essential oil production; specifically, those rich in 1,8-cineole are highly sought after in the pharmaceutical and cosmetic industries [4,5]. This genus is divided into 13 subgenera, with around 800 species and subspecies identified. The largest subgenera is ‘*Symphyomyrtus*’, containing over 500 species, some of which have industrial importance due to their phytochemical contents in the essential oils. For instance, there are groups of species with high essential oil yields, highlighting their economic importance, and 1,8-cineole as the major component, which possesses various medicinal properties, including antibacterial and antifungal activities: *E. globulus*, *E. polybractea*, *E. camaldulensis*, etc. [4,6,7]. A systematic review of 306 studies by [3] indicated that Eucalyptus essential oils showed potential in treating respiratory disorders and cancer, where 1,8-cineole exhibits anti-inflammatory properties beneficial for several diseases. There are several reviews, most of which are focused on the aforementioned species, that summarize ethnopharmacological uses, phytochemical contents, pharmacological activities and possible reaction mechanisms from time to time [3,8,9,10].

The details of historical records of the genus *Eucalyptus* in Northern Cyprus are given in a book published by the Chamber of Forest Engineers of Northern Cyprus [11]. Eucalypts were first introduced to Cyprus in 1876 by the French arborist P.G. Madon who was appointed by the Ottoman government to prepare a report on the existing forests of the island. Madon published his first report titled *Replanting of the island of Cyprus* in 1880, followed by a second report in 1881, which focused on the preservation of the forests. During this period, the eucalyptus was seen as a potential solution to the deforestation issues in Cyprus, particularly in areas like the Mesaoria Plain and the Kyrenia Mountains, which were noted to be largely bare. The introduction of eucalyptus was part of broader efforts to improve the ecological conditions in Cyprus, similar to its use in Algeria, where it was effective in combating malaria by draining the swamps [11,12]. Since 2014, it has been reported that approximately sixty-two species have been naturalized, which includes six widespread species *E. camaldulensis* (var. *camaldulensis* and var. *obtusa*), *E. gomphocephala*, *E. torquata*, *E. sargentii*, *E. astringens* and *E. occidentalis* [11]. The ethnobotanical usage of this genus is limited. The leaves of *E. camaldulensis* are utilized for various medicinal purposes, such as treating respiratory and musculoskeletal disorders, while *E. torquata* lacks documented ethnobotanical uses in Cyprus [2,13,14].

Studies on the essential oil composition of *E. torquata*, belonging to the largest subgenera of the genus *Eucalyptus*, *Symphyomyrtus*, were limited regarding their distribution around the world [2,5,15,16,17]. The main compounds in the leaf essential oil of *E. torquata* collected from Cyprus were α-pinene (18.6%), 1,8-cineole (18.8%), β-eudesmol (10.3%) and torquatone (29.2%) [2]. In a study on the essential oil compositions of the samples from Australia belonging to the subgenera *Symphyomyrtus*, section *Dumaria*, series *Torquatae*, which also includes *E. torquata*, reported that α-pinene (18.79%), torquatone (40.91%). It was also reported that the ten separate analyses of the same tree during two years period revealed torquatone valued 37.9 ±3.4% while the samples collected from ten different trees located in different places showed torquatone valued 35.0 ±3.5% [18]. Torquatone together with isotorquatone, miniatone, make up the class of acylphloroglucinols, found in the Myrtaceae family, which are important for the genus *Eucalyptus*, all subgenera of which present the acylphloroglucinol class [6,18,19]. The major constituents of a Tunisian sample was α-pinene (10.5%), 1,8-cineole (12.0%), β-eudesmol (10.1%) and torquatone (42.0%) [5]. In a study on the same species cultivated in Iran, the essential oil of fresh leaves was dominated by 1,8-cineole (66.9%), α-pinene (13.9%) and *trans*-pinocarveol (6.3%) [17]. In another research, the main compounds of the essential oil of leaves collected from Iran were reported as 1,8-cineole (69.6%), α-pinene (9.5%), aromadendrene (4.5%) and alloaromadendrene (7.8%) [16]. In another study, the major compounds of the essential oil were reported as 1,8-cineole (28.6%), α-pinene (15.7%) and globulol (13.1%) [15].

The aim of this study is to investigate the phytochemical compositions of the essential oils obtained separately from both juvenile and mature leaves for a period of a year from the same tree in Nicosia/Cyprus. Multivariate statistical analysis was also conducted to show the possible temporal variations between seasonal essential oils of both types of leaves.

## 2. Results and Discussion

Table 1 gives information about the codes, collection dates and the essential oil yields of juvenile and mature leaves belonging to a one-year period, while Table 2 and Table 3 reveal information about the essential oil compositions of juvenile and mature leaves over a 12-month period.

The essential oil yields were in the range of 1.00–3.18% and 0.53–3.24% for the mature leaves and juvenile leaves, respectively. The highest yields were obtained in December (3.18%) for mature leaves and in November (3.24%) for juvenile leaves, which were as high as one of the highest essential oil yields reported for this species from Tunisia at 3.2 ± 0.4% [5].

**Table 1:** The collection dates and essential oil yields of *E. torquata* samples.

Code	Collection Date	Essential Oil Yields (%, *v*/*w*)	
		Juvenile Leaves	Mature Leaves
Jan	13 Janaury 2023	1.56	1.50
Feb	12 February 2023	0.96	2.00
Mar	15 March 2023	0.53	2.00
Apr	10 April 2023	0.91	1.00
May	11 May 2023	2.59	1.00
Jun	13 June 2023	1.71	1.60
Jul	11 July 2023	1.08	2.00
Aug	10 August 2023	2.36	1.00
Sep	10 September 2023	3.12	2.00
Oct	10 October 2023	2.77	1.60
Nov	10 November 2023	3.24	1.80
Dec	10 December 2023	1.81	3.18

The major compounds of the essential oils of the mature leaves over the course of 12 months were torquatone (27.6–48.8%) and 1,8-cineole (3.6–27.8%), followed by α-pinene (8.1–12.9%), β-eudesmol (7.9–12.9%) and α-eudesmol (3.1–8.4%), whereas the major compounds of the essential oils of the juvenile leaves over 12 months were torquatone (28.8–53.2%) and 1,8-cineole (4.9–22.7%). In addition, the other important compounds were α-pinene (8.8–23.1%), β-eudesmol (4.0–8.6%) and α-eudesmol (3.8–7.6%). Even though the torquatone level reached almost half the essential oil content in some of the months in the present study’s results, the percentages of this compound in the leaf essential oils of the same species reported in the previous literature were quite variable; in Iran and Morocco, samples were absent of this chemical [15,16,17,20,21], whereas it was 29% in Cyprus [2] and 42% in Australia and Tunisia samples [5,22]. The major compounds of the essential oil of *E.torquata* previously reported in the literature are summarized in Table 4.

**Table 2:** The essential oil compositions for a 12-month period of *E. torquata* juvenile leaves.

LRI Lit.	LRI PS	Compound Name	Relative Percentage Amounts in Juvenile Leaves (%)											
			Jan	Feb	Mar	Apr	May	Jun	Jul	Agu	Sep	Oct	Nov	Dec
1008–1039 ^b^	1015	α-Pinene	8.8	17.9	10.3	17.4	15.4	21.7	22.7	15.8	14.8	13.7	23.1	13.3
1043–1086 ^b^	1035	Camphene	-	-	-	0.1	tr	-	0.1	tr	0.1	tr	-	tr
1085–1130 ^b^	1082	β-Pinene	1.0	0.6	0.3	0.4	0.4	0.6	0.5	0.5	0.4	0.4	0.7	0.4
1140–1175 ^b^	1136	Myrcene	0.2	0.3	0.2	0.2	0.2	0.3	0.1	0.3	0.2	0.2	0.3	0.2
1148–1186 ^b^	1140	α-Phellandrene	0.8	0.6	0.2	0.2	0.3	0.2	0.1	0.6	0.6	0.7	0.4	0.3
1154–1195 ^b^	1155	α-Terpinene *	-	0.1	-	-	-	-	-	0.1	0.1	tr	-	tr
1178–1219 ^b^	1173	Limonene	1.2	1.4	0.8	1.1	1.0	1.3	1.0	1.1	1.0	1.1	1.3	1.0
1188–1233 ^b^	1184	β-Phellandrene	-	-	-	-	0.1	-	-	0.1	0.1	0.1	-	tr
1186–1231 ^b^	1186	1,8-Cineole	15.4	22.7	4.9	13.0	14.8	20.2	12.7	13.7	11.9	16.7	21.5	14.8
1222–1266 ^b^	1218	γ-Terpinene	-	0.1	0.1	0.1	0.1	0.1	-	0.1	0.1	0.1	0.1	0.1
1246–1291 ^b^	1246	p-Cymene	0.6	0.4	0.3	0.3	0.3	0.4	0.6	0.5	0.4	0.6	0.2	0.3
1261–1300 ^b^	1256	Terpinolene	-	0.1	0.1	0.1	0.1	0.1	0.1	0.1	0.1	0.1	0.1	0.1
1511–1545 ^b^	1497	α-Gurjunene *	0.4	0.2	0.1	0.5	0.2	0.2	0.3	0.2	0.1	0.1	0.2	0.1
1545–1590 ^b^	1551	Pinocarvone	0.3	0.1	tr	0.3	0.2	0.2	0.5	0.1	0.1	0.1	0.3	0.1
1564–1618 ^b^	1562	β-Gurjunene *	-	-	-	0.1	tr	-	0.1	-	tr	tr	-	tr
1570–1685 ^b^	1568	β-Caryophyllene	-	-	-	-	0.1	0.1	tr	-	0.1	tr	-	tr
1564–1630 ^b^	1573	Terpinen-4-ol	-	0.2	0.1	0.4	0.2	0.2	0.3	0.1	0.1	0.1	0.2	0.1
1583–1668 ^b^	1577	Aromadendrene	3.2	1.1	0.8	3.8	1.2	1.6	3.5	0.9	0.6	0.4	1.4	0.6
	1585	Selina-5,11-diene *	-	-	-	0.1	0.1		0.1	tr	tr	tr	0.2	tr
1624–1668 ^b^	1616	Alloaromadendrene	0.3	0.1	0.1	0.4	0.2	0.2	0.3	0.1	0.1	0.1	-	0.1
1643–1671 ^b^	1629	*trans*-Pinocarveol	0.9	0.2	0.1	0.6	0.5	0.6	1.7	0.1	0.2	0.4	0.7	0.2
1637–1689 ^b^	1641	α-Humulene *	-	-	-	-	tr	-	tr	tr	tr	tr	-	tr
1620–1679 ^a^	1658	*E*-Methyl geranate *	-	-	-	-	0.1	-	tr	tr	-	tr	-	0.1
1656–1707 ^a^	1663	Ledene *	0.4	0.3	0.2	0.5	0.2	0.2	0.3	0.2	0.2	0.1	0.2	0.2
1629–1724 ^b^	1665	α-Terpineol	0.9	0.7	0.4	1.1	0.5	0.6	0.9	0.5	0.4	0.3	0.5	0.4
1653–1728 ^b^	1672	Borneol	0.3	0.1	0.1	0.2	0.1	0.1	0.2	0.1	0.1	0.1	0.1	0.1
1688–1761 ^b^	1691	Valencene *	-	0.1	-	0.1	0.1	0.1	0.1	0.1	0.1	0.1	-	0.1
1664–1688 ^a^	1694	Selina-4,11-diene *	-	-	-	0.1	tr	0.1	tr	0.1	0.1	tr	-	tr
1704–1737 ^a^	1697	β-Dihydro agarofuran *	-	0.1	-	tr	0.1	0.1	tr	0.1	0.1	0.1	-	0.1
1840–1949 ^b^	1711	Piperitone	-	0.1	-	-	tr	-	tr	tr	tr	tr	-	tr
1722–1774 ^b^	1720	δ-Cadinene *	-	0.1	-	0.1	0.1	0.1	0.1	0.1	0.1	tr	-	tr
1743–1808 ^b^	1762	Myrtenol	-	0.1	-	-	0.1	tr	0.1	tr	-	tr	-	0.1
1790–1886 ^a^	1770	Calamenene *	-	-	-	0.1	tr	0.1	0.1	tr	tr	tr	-	tr
	1893	5,11-Epoxy-1(10)-cadinene *	-	-	-	0.1	tr	-	0.1	tr	-	-	-	-
1911–1938 ^b^	1904	Palustrol	-	-	-	0.1	tr	tr	0.1	tr	tr	tr	-	tr
	1969	Amyl phenyl acetate *	-	-	-	0.1	tr	0.1	0.1	tr	-	tr	-	tr
1973 ^a^	1974	Maaliol	0.3	0.1	0.1	0.2	0.1	0.1	0.2	0.1	0.1	0.1	0.1	0.1
2025–2033 ^a^	1982	*epi*-globulol	1.1	0.5	0.5	1.1	0.4	0.4	0.9	0.5	0.3	0.3	0.4	0.4
2014–2062 ^b^	2006	Ledol	-	0.1	0.1	0.2	0.1	0.1	0.1	0.1	0.1	0.1	0.1	0.1
2061–2074 ^a^	2025	Cubeban-11-ol	0.3	0.1	0.2	0.3	0.1	0.1	0.2	0.1	0.1	0.1	0.1	0.1
2065 ^a^	2043	10-*epi*-elemol *	-	-	-	-	tr	-	tr	-	0.1	0.1	-	0.1
2049–2104 ^b^	2049	Globulol	4.4	2.3	2.6	4.7	1.8	1.6	4.0	2.2	1.4	1.5	1.7	1.9
2041–2110 ^b^	2057	Viridiflorol	0.6	0.3	0.4	0.7	0.3	0.3	0.5	0.3	0.2	0.2	0.3	0.3
2133–2144 ^a^	2085	Rosifoliol	0.4	0.4	0.6	0.7	0.4	0.3	0.6	0.4	0.3	0.3	0.3	0.3
	2117	Muurola-4,10(14)-diene-1-ol *	-	-	-	0.1	0.1	-	tr	-	-	tr	0.1	tr
2147–2199 ^b^	2135	γ-Eudesmol	3.6	2.0	3.0	2.5	2.9	2.2	2.2	3.0	3.9	3.0	1.9	3.2
2178–2204 ^a^	2147	Eremoligenol	1.8	0.8	1.2	1.0	1.0	0.7	0.8	1.0	1.1	1.1	0.7	1.2
2081–2108 ^a^	2157	α-Guaiol	1.1	0.5	0.8	0.6	0.7	0.5	0.5	0.6	0.8	0.8	0.4	0.8
2140–2246 ^b^	2170	Carvacrol	-	-	-	-	0.1	-	tr	-	-	tr	0.2	0.1
	2176	Agarospirol *	-	-	-	-	tr	-	tr	-	-	tr	-	-
2215–2231 ^a^	2184	Valerianol	-	0.1	0.1	0.1	0.1	-	0.1	0.1	0.1	0.1	0.1	-
2186–2250 ^b^	2194	α-Eudesmol	7.6	3.9	6.6	4.7	5.7	4.2	3.8	5.5	7.4	6.6	3.9	6.9
2180–2255 ^b^	2199	α-Cadinol *		0.1	0.1	0.1	0.1	0.1	0.1	0.1	0.1	0.1	0.1	0.1
2196–2272 ^b^	2205	β-Eudesmol	8.6	4.0	7.4	4.9	6.2	4.5	4.9	5.7	8.0	7.8	4.2	7.4
2207–2274 ^b^	2226	Selin-11-en-4-a-ol	-	0.1	0.2	0.2	0.1	0.1	0.1	0.1	0.1	0.1	0.1	0.1
2286 ^c^	2244	Apodophyllone	0.5	0.6	1.1	0.3	0.6	0.2	0.2	0.6	1.0	1.0	0.3	1.2
2278–2387 ^b^	2305	(*E*,*E*)-Farnesol *	-	-	0.1	0.1	0.1	0.1	0.1	0.1	0.1	0.1	-	0.1
2379 ^c^	2334	Isotorquatone	-	0.4	0.6	0.4	0.4	0.3	0.3	-	0.5	0.4	0.3	0.5
2424 ^a^	2376	Torquatone	32.1	34.5	53.2	30.6	38.9	32.7	28.8	41.5	40.7	39.1	31.2	40.6
2518 ^c^	2482	Miniatone	1.7	1.2	1.6	4.0	2.3	2.1	3.1	1.4	1.0	0.6	2.0	1.0
	Total		99.0	99.6	99.6	98.9	99.1	99.6	99.0	99.2	99.6	99.6	99.6	99.6

LRI Lit.: The LRI from the literature reported for PEG-based columns (INNOWAX, DB-WAX, HP-WAX, Carbowax etc.) [23] ^a^, [24] ^b^, [25] ^c^, LRI PS: Linear retention indices calculated against *n*-alkanes, % calculated from FID data in the present study, tr: Trace < 0.1. * The ones that only exist in juvenile leaf essential oils, excluded from PCA and HCA.

**Table 3:** The essential oil compositions for a 12-month period of *E. torquata* mature leaves.

LRI Lit.	LRI PS	Compound Name	Relative Percentage Amounts in Mature Leaves (%)											
			Jan	Feb	Mar	Apr	May	Jun	Jul	Agu	Sep	Oct	Nov	Dec
1008–1039 ^b^	1015	α-Pinene	12.9	11.1	11.9	10.4	8.9	8.1	10.3	13.5	9.2	12.0	12.1	9.7
1043–1086 ^b^	1035	Camphene	tr	-	tr	tr	-	tr	tr	tr	tr	0.1	tr	tr
1085–1130 ^b^	1082	β-Pinene	0.3	0.3	0.3	0.3	0.2	0.3	0.3	0.4	0.3	0.2	0.4	0.3
1101–1136 ^a^	1101	Isoamyl acetate *	0.1	0.1	0.1	0.1	-	0.1	tr	0.1	0.1	0.1	0.2	tr
1140–1175 ^b^	1136	Myrcene	0.2	0.1	0.2	0.1	0.1	0.1	tr	0.2	0.1	0.1	0.2	0.1
1148–1186 ^b^	1140	α-Phellandrene	0.8	0.2	0.9	0.1	0.2	0.2	0.1	1.2	0.3	0.1	0.6	0.8
1154–1195 ^b^	1155	α-Terpinene *	0.1	-	0.1	-	-	tr	-	0.1	tr	tr	tr	0.1
1178–1219 ^b^	1173	Limonene	1.0	0.7	0.9	0.7	0.6	0.6	0.7	1.0	0.6	0.9	1.1	0.7
1188–1233 ^b^	1184	β-Phellandrene	0.1	0.1	0.1	0.1	-	0.1	0.1	0.1	tr	0.1	0.1	0.1
1186–1231 ^b^	1186	1,8-Cineole	15.2	18.0	15.6	14.3	3.6	13.3	12.5	13.1	8.1	27.8	26.2	9.9
1222–1266 ^b^	1218	γ-Terpinene	0.1	0.1	0.1	0.1	0.1	0.1	0.1	0.1	0.1	tr	0.1	0.1
1246–1291 ^b^	1246	*p*-Cymene	1.6	1.8	1.4	1.4	0.6	1.7	2.5	0.8	1.3	2.3	1.1	0.5
1261–1300 ^b^	1256	Terpinolene	tr	-	tr	tr	-	tr	tr	0.1	tr	tr	0.1	0.1
1277–1304 ^a^	1268	Isoamyl isovalerate *	0.1	0.2	0.1	0.1	0.1	0.1	tr	0.1	0.1	0.1	0.1	0.1
1380–421 ^a^	1343	2-Octanol *	tr	-	tr	tr	-	tr	0.1	tr	tr	0.1	0.1	tr
	1519	1-isopropyl-3-methyl butyl acetate *	0.1	-	0.1	0.1	-	0.1	1.3	0.1	0.1	0.1	0.3	0.1
1545–1590 ^b^	1553	Pinocarvone	0.6	1.6	0.7	1.1	0.2	1.0	-	0.3	0.4	1.7	0.5	0.2
1570–1685 ^b^	1570	β-Caryophyllene	0.1	-	0.1	tr	-	tr	-	0.1	tr	0.1	0.1	0.1
1564–1630 ^b^	1575	Terpinen-4-ol	0.1	-	0.1	0.1	-	0.1	0.1	0.2	0.1	0.1	0.2	0.2
1583–1668 ^b^	1579	Aromadendrene	0.3	0.3	0.3	0.3	0.4	0.3	0.2	0.7	0.7	0.5	0.7	1.0
1624–1668 ^b^	1617	Alloaromadendrene	0.1	-	0.1	tr	0.1	tr	-	0.1	0.1	0.1	0.1	0.1
1643–1671 ^b^	1632	*trans*-Pinocarveol	2.0	4.8	2.3	3.5	0.6	3.1	4.2	0.8	1.5	5.4	1.5	0.7
1662–1717 ^b^	1658	Limonene-4-ol *	tr	-	tr	0.1	-	tr	0.1	tr	0.1	0.1	0.1	-
1686–1697 ^a^	1664	Carvotan acetone *	0.1		0.1	0.1	-	0.1	0.1	0.2	0.1	0.1	0.1	0.2
1629–1724 ^b^	1667	α-Terpineol	0.2	0.3	0.2	0.3	0.2	0.2	0.5	0.3	0.4	0.5	0.3	0.2
1653–1728 ^b^	1675	Borneol	0.1	0.2	0.1	0.2	0.1	0.1	0.2	0.1	0.1	0.3	0.1	0.1
	1699	2-Acetoxy-1,8-cineole *	0.1	0.1	0.1	0.1	0.1	0.1	0.1	0.1	0.1	0.1	0.1	0.1
1840–1949 ^b^	1713	Piperitone	0.1	0.1	0.1	0.1	-	0.1	0.1	0.1	0.1	0.1	0.1	0.1
1743–1808 ^b^	1764	Myrtenol	0.1	0.2	0.1	0.1	-	0.1	0.1	tr	0.1	0.1	0.1	tr
1810–1821 ^a^	1767	*trans*-*p*-mentha-1,(7),8-diene-2-ol *	0.1	0.1	0.1	0.1	-	0.1	0.1	tr	tr	0.2	0.1	tr
	1779	*p*-mentha-1,(7),5-diene-2-ol *	0.1	-	0.1	tr	-	0.1	0.1	0.1	tr	0.1	0.1	tr
1801–1879 ^a^	1803	*trans*-Carveol *	0.1	0.2	0.1	0.1	-	0.1	0.2	tr	0.1	0.2	0.1	tr
1820–1881 ^a^	1818	*p*-Cymene-8-ol *	tr	0.1	tr	0.1	-	tr	0.1	tr	tr	0.1	tr	tr
1885–1903 ^a^	1857	*cis*-*p*-mentha-1,(7),8-diene-2-ol *	0.1	0.1	0.1	0.1	-	0.1	0.1	tr	0.1	0.2	0.1	tr
	1874	α-Phellandrene epoxide *	0.1	-	0.1	tr	-	0.1	0.1	tr	tr	0.1	tr	-
1911–1938 ^b^	1907	Palustrol	tr	-	tr	tr	-	tr	tr	0.1	0.1	tr	tr	0.1
1973 ^a^	1977	Maaliol	0.1	0.1	0.1	0.1	0.1	0.1	0.1	0.1	0.1	0.1	0.1	0.1
2025–2033 ^a^	1984	*epi*-globulol	0.3	0.3	0.3	0.4	0.5	0.4	0.2	0.4	0.4	0.3	0.3	0.4
2014–2062 ^b^	2008	Ledol	0.1	0.1	tr	0.1	0.1	0.1	0.1	0.1	0.1	0.1	0.1	0.1
2061–2074 ^a^	2027	Cubeban-11-ol	0.1	0.1	0.1	0.1	0.2	0.1	0.1	0.1	0.1	0.1	0.1	0.1
2043–2013 ^b^	2046	Elemol *	0.1	0.1	0.1	0.1	0.1	0.1	0.1	0.1	0.1	0.1	0.1	0.1
2049–2104 ^b^	2052	Globulol	1.5	1.9	1.5	2.1	2.5	2.0	1.3	2.0	2.0	1.4	1.6	1.9
2041–2110 ^b^	2059	Viridiflorol	0.2	0.3	0.2	0.3	0.4	0.3	0.2	0.3	0.3	0.2	0.3	0.3
2133–2144 ^a^	2079	Rosifoliol	0.1	0.1	0.1	0.1	0.2	0.1	0.1	0.2	0.1	0.1	0.1	0.2
	2088	α-Thujaplicin *	0.4	0.2	0.2	0.2	-	0.2	0.2	0.4	-	-	-	-
2074–2150 ^b^	2096	Spathulenol *	0.2	0.3	0.2	0.2	0.2	0.2	0.2	0.4	0.4	0.1	0.2	0.2
2147–2199 ^b^	2138	γ-Eudesmol	2.8	1.2	2.7	1.6	2.8	1.8	1.4	3.5	2.0	0.7	2.4	2.8
2178–2204 ^a^	2150	Eremoligenol	1.6	1.2	1.5	1.3	1.9	1.4	1.3	1.8	1.8	1.0	1.3	1.6
2081–2108 ^a^	2160	α-Guaiol	1.0	0.7	1.0	0.8	1.2	0.9	0.7	1.1	1.1	0.5	0.9	1.0
2140–2246 ^b^	2173	Carvacrol	0.1	0.1	0.1	0.1	0.1	0.2	0.2	0.1	0.2	0.2	0.1	0.1
	2187	Valerianol	0.1	0.1	0.1	0.1	0.2	0.1	0.2	0.2	0.2	0.1	0.1	0.2
2186–2250 ^b^	2197	α-Eudesmol	7.5	5.0	7.5	5.3	8.4	6.1	4.7	7.9	6.8	3.1	6.5	7.3
2196–2272	2207	β-Eudesmol	10.0	9.8	10.2	10.3	12.9	10.9	10.9	9.7	11.6	7.9	8.5	9.9
2207–2274 ^b^	2229	Selin-11-en-4-α-ol	0.1	0.2	0.1	0.1	0.2	0.2	0.1	0.1	0.1	0.1	0.1	0.1
2286 ^c^	2256	Apodophyllone	1.0	1.0	1.0	0.9	1.2	0.7	0.5	0.6	1.1	0.9	0.8	0.7
2379 ^c^	2337	Isotorquatone	0.4	0.5	0.4	0.5	0.7	0.4	0.3	0.4	0.4	0.4	0.3	0.4
2424 ^a^	2378	Torquatone	34.6	35.1	35.5	39.9	48.8	41.9	41.4	34.8	45.1	27.6	28.0	45.2
2518 ^c^	2484	Miniatone	0.6	0.5	0.6	0.6	0.9	0.8	1.1	0.9	0.8	0.5	0.6	1.1
	Total		99.7	99.7	99.8	99.7	99.5	99.7	99.9	99.8	99.3	99.5	99.5	99.4

LRI Lit.: The LRI from the literature reported for PEG-based columns (INNOWAX, DB-WAX, HP-WAX, Carbowax etc.) [23] ^a^, [24] ^b^, [25] ^c^, LRI PS: Linear retention indices calculated against *n*-alkanes, % calculated from FID data in the present study, tr: Trace < 0.1. * The ones that only exist in mature leaf essential oils, excluded from PCA and HCA.

**Table 4:** The major components of the essential oils of *E. torquata* reported previously in the literature.

Country	Major Compounds (%)	Reference
Cyprus	α-pinene (18.6%), 1,8-cineole (18.8%), β-eudesmol (10.3%), torquatone (29.2%)	[2]
Tunisia	α-pinene (10.5%), 1,8-cineole (12.0%), β-eudesmol (10.1%), torquatone (42.0%)	[5]
Iran	1,8-cineole (28.6%), α-pinene (15.7%), globulol (13.1%)	[15]
Iran	1,8-cineole (69.6%), α-pinene (9.5%), aromadendrene (4.5%), alloaromadendrene (7.8%)	[16]
Iran	1,8-cineole (66.9%), α-pinene (13.9%), trans-pinocarveol (6.3%)	[17]
Australia	α-pinene (18.79%), torquatone (40.91%)	[18]
Iran	α-pinene (20.0%), 1,8-cineole (24.2%), globulol (8.4%), aromadendrene (7.8%)	[20]
Morocco	α-pinene (16.7 and 20.0%), 1,8-cineole (46.9 and 28.9%) borneol (10.8 and 22.6%)	[21]
Australia	Torquatone (42.0%), 1,8-cineole (11.2%), α-pinene (10.2%), α-eudesmol (10.2%), β-eudesmol (11.1%), γ-eudesmol (4.8%)	[22]

Correlation analyses were run between the characteristic or major constituents of the essential oils, specifically torquatone, 1,8-cineole, α-eudesmol, β-eudesmol and α-pinene. Torquatone was found to have pronounced negative correlations with 1,8-cineole, α-eudesmol, β-eudesmol and α-pinene (*p* < 0.001, *p* = 0.021, *p* = 0.012 and *p* = 0.007, respectively).

**Figure 1:**
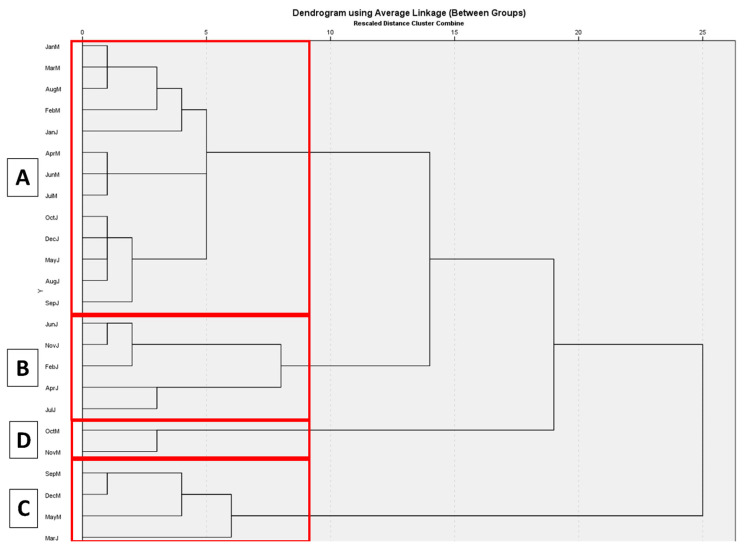
The squared Euclidean distance HCA dendrogram of the essential oils of juvenile and mature leaves collected during the one-year period. (**A**–**D**) denote different compositional clusters.

The squared Euclidean distance HCA dendrogram of the essential oils of juvenile and mature leaves collected during the one-year period is given in Figure 1. The cutoff distance at the nearest integer between clusters was determined to be 9. The HCA revealed the existence of three clusters (A–C) and one pseudocluster (D) which comprised mature leave samples belonging to October and November. The demarcation between a true cluster and pseudocluster was taken to be the existence of at least three samples per cluster. As a result, D was excluded from further analysis due to failure to meet minimum sample number criterion. A complex clustering pattern was observed between the mature and juvenile sample groups, whereby the entirety of Cluster B and the majority of Cluster C were composed of mature leaves, but Cluster A, the largest, was composed of an approximately equal number of juvenile and mature leaves.

Based on the clustering results, the current dataset does not support the notion that mature and juvenile leaves have distinctly different evolutions of their composition over a 12-month period, but they are instead subject to complex composition evolution, yielding significant temporal variance in the composition of essential contents in leaves that requires detailed analysis.

**Figure 2:**
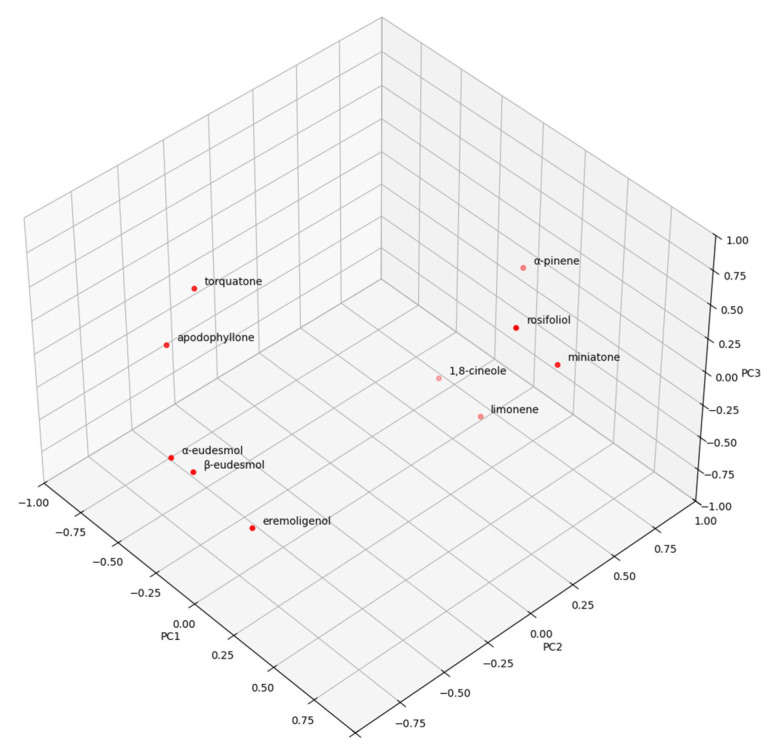
The PCA results of the first three principal components.

Principal Component Analysis results produced a Scree Plot consistent with the existence of nine principal components, with strong correlations with certain phytochemicals (Figure 2). These nine principal components were found to be capable of explaining a total of 97.843% of the variation observed in the samples; therefore, these nine principal components were considered to be definitive in the temporal evolution of distinct compositions.

These indicate the presence of a complex interplay between a large number of phytochemicals, projecting the existence of a complex identification profile for chemical composition. Nevertheless, these phytochemicals were selected and subjected to ANOVA of their preponderance across the clusters determined by HCA to elucidate any statistically significant differences. Certain phytochemicals were found to act as chemical biomarkers to distinguish between the groups. The results of the ANOVA analysis confirmed the existence of three clusters, named A through C. The analysis of the major compounds in all samples revealed that Cluster C has the highest mean torquatone concentration (48.07%), followed by Clusters A (38.17%) and B (31.56%). 1,8-cineole mean concentrations in Clusters A (14.57%) and B (18.02%) were statistically similar but higher than the other cluster (6.64%).

The analyses of the other important compounds regarding their relative percentage amounts in the essential oils of all samples showed the following results. α-eudesmol was present in higher concentrations in Clusters A (6.43%) and C (7.28%) than B (4.11%), and β-eudesmol presented with a similar profile, presenting higher concentrations in Cluster A (8.89%) and C (10.45%) which were determined to be elevated compared to B (4.48%). α-pinene concentrations showed a gradual decrease between Clusters B (20.55%), A (12.31%) and C (9.54%), in order. Among the taxospecific acylphloroglucinols, isotorquatone was observed to have no statistically significant differences between the clusters, whereas miniatone was present with a statistically important enrichment in Cluster B and was present in poorer equivalent concentrations in Clusters A and C.

The ANOVA conducted on the clusters also determined distinguishing characteristics between the clusters for relatively minor compounds. The compounds and their mean values in clusters are limonene (0.8–1.4%, juvenile leaves; 0.6–1.1%, mature leaves), apodophyllone (0.2–1.2%, juvenile leaves; 0.5–1.2%, mature leaves) and α-terpineol (0.3–1.1%, juvenile leaves; 0.2–0.5%, mature leaves) whose concentrations were significantly higher in Cluster B, but no significant differences were observed between A and C. On the other hand, eremoligenol (0.7–1.8%, juvenile leaves; 1.0–1.9%, mature leaves) was highly present in Cluster A and C compared to Cluster B.

The statistical analysis for the comparison of the leaf essential oil components between juvenile and mature leaves demonstrated that there is a significant difference in the concentration of one of the major compounds, α-pinene, higher in juvenile essential oils (*p* < 0.001) over the course of a year. On the other hand, β-eudesmol, another major compound, was found at a significantly higher concentration (*p* < 0.001) in the mature leaf essential oils. The other major compounds were not observed with statistically significant differences in concentration. However, only miniatone was found at significantly higher concentrations (*p* < 0.002) in the essential oils of juvenile leaves. Torquatone and isotorquatone were not found to be different in concentration between mature and juvenile leaves. *p*-cymene, pinocarvone and *trans*-pinocarveol were all found to be significantly higher in mature leaves than juveniles (*p* < 0.001, *p* = 0.011 and *p* < 0.001, respectively). In addition, limonene and rosifoliol were present at higher concentrations in juvenile leaves (*p* < 0.001), with reduced concentrations in mature leaves.

**Figure 3:**
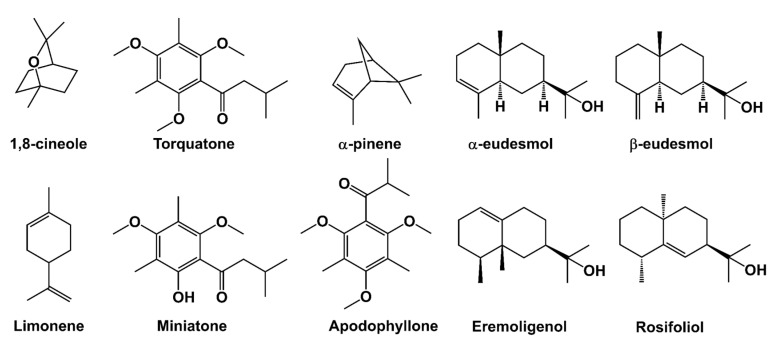
The skeletal formulae of the major constituents and other important compounds, as well as statistically relevant constituents.

**Figure 4:**
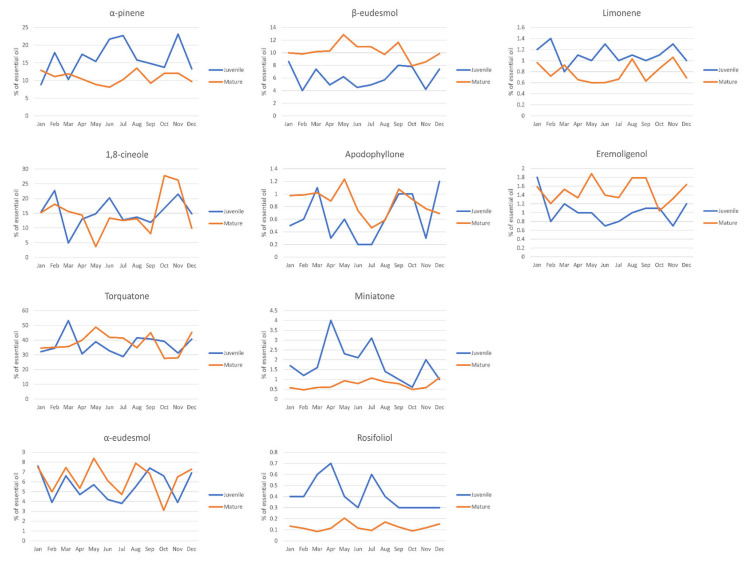
The temporal evolution of major, important and statistically significant constituents in mature and juvenile leaf essential oils.

The skeletal formulae of the major constituents, as well as statistically relevant constituents, are given in Figure 3. In the literature, the relevant NMR data of some of those molecules, namely apodophyllone, torquatone, miniatone and some other aryl ketones, found in the essential oil of *Eucalyptus* sp., have been given [25]. The temporal evolutions of major, important and statistically significant constituents in mature and juvenile leaf essential oils are provided in Figure 4.

Regression analysis was conducted to determine if certain edaphic factors, namely yearly sunshine hours, yearly precipitation and monthly average temperatures, contribute to the trends in temporal change in composition. The compounds of the essential oils of mature leaves in the all-month period revealed a time-dependent increase in aromadendrene and alloaromadendrene concentrations in the quadratic regime (0.008x^2^ + 0.052x + 0.372 and 0.001x^2^ − 0.011x + 0.055, respectively, where x denotes the numeric order of the months). In either case, no coefficient was found to be dominant. Other phytochemicals were not found to fit quadratic or cubic models. In addition, the compounds of essential oils of mature leaves versus the sunshine showed that there is no relation between the majority of the compounds. Among the phytochemicals that were observed to have a relationship with sunlight were eremoligenol and β-eudesmol at a constant concentration in cubic modeling (*p* = 0.034 and *p* = 0.006). Apodophyllone was found to have a true dependence on sunlight (*p* = 0.050), peaking at low sunlight levels and reducing at higher illumination.

## 3. Materials and Method

### 3.1. Collection of Plant Samples

*Eucalyptus* sp. is known for its heterophyllous leaves; in *E. torquata*, both juvenile and mature leaves are petiolate and alternate. Juvenile leaves are elliptic or lanceolate, dark green, 9 cm in length and 2.5 to 3 cm in width, while mature leaves are 9 to 13 cm in length and 1.3 to 2 cm in width, narrowly lanceolate and matte-green- or grayish-green-colored. The flowering season is from March to April, and the fructification season is from August to November [11]. The juvenile and mature leaves of *E. torquata* were collected for 12 months approximately on the same date and time from the same tree located in Near East University Campus, Nicosia, Cyprus. Identification of the collected *Eucalyptus* species was carried out by Prof. Dr. Dudu Özkum Yavuz, Assist. Prof. Dr. Duygu Yiğit Hanoğlu and Assist. Prof. Dr. Azmi Hanoğlu. The voucher specimens were kept at the Near East University herbarium (NEUN) with the number of NEUN20009.

### 3.2. Isolation of the Essential Oil

The air-dried juvenile and mature leaves (50–100 g) of the *E. torquata* samples were gently crushed into tiny pieces and were separately hydrodistilled for 3 h, using a *Clevenger*-type apparatus. The resulting essential oils were collected in amber vials and stored at 4 °C until the analysis. Table 1 tabulates information about the sample codes, collection dates and essential oil yields of all plant materials.

### 3.3. Gas Chromatography

GC analysis is carried out using an Agilent 7890B GC system (Agilent Technologies, Santa Clara, CA, USA). The FID detector temperature is 300 °C. To obtain the elution order with GC-MS, simultaneous auto-injection is performed on a duplicate of the same column applying the same operational conditions. Relative percentage amounts of the separated compounds were calculated from FID chromatograms.

### 3.4. Gas Chromatography/Mass Spectrometry (GC/MS)

The GC-MS analysis is carried out using an Agilent 5977B GC-MSD system. Innowax FSC column (60 m × 0.25 mm, 0.25 μm film thickness) was used with helium as a carrier gas (0.8 mL/min). GC oven temperature was kept at 60 °C for 10 min and programmed to 220 °C at a rate of 4 °C/min, and kept constant at 220 °C for 10 min and then programmed to 240 °C at a rate of 1 °C/min. The split ratio was adjusted at 40:1. The injector temperature was set at 250 °C. Mass spectra were recorded at 70 eV. The mass range was from *m/z* 35 to 450.

### 3.5. Identification of the Constituents

This was carried out by comparing the relative retention times of the essential oil constituents with those of authentic samples or by comparisons of their linear retention index (LRI) to a series of *n*-alkanes (C_8_–C_40_). Computer matching against commercial (Wiley GC/MS Library, NIST Chemistry WebBook) [23,26] and in-house ‘Başer Library of Essential Oil Constituents’ built up by genuine compounds and components of known essential oils, as well as MS literature data were used for the identification [27].

### 3.6. Statistical Analysis

All relevant data were imported to IBM SPSS Statistics v27.0 (International Business Machines (IBM) Corporation, Chicago, IL, USA). Principal Component Analysis (PCA) was performed using the Correlation Matrix method (Supplementary Materials) with sequential eigenvalues selected based on the introduction of “kinks” in the Scree Plot. The Varimax Rotation method was employed to improve the correlation between chemical constituents and principal components. Correlation Matrices were employed to ascertain the effect of different constituents on chemical composition. Hierarchical Cluster Analysis (HCA) was performed using the Squared Euclidean Distance Between-Groups Linkage method using agglomeration schedules. The dendrograms were produced from the HCA using these data. Only components that were deemed major by the authors of at least 1 of the references cited herein were included in the PCA and HCA. The clades as determined by HCA were subjected to one-way Analysis of Variance (ANOVA) analysis to confirm chemical differences, with Levene’s test employed to test for homogeneity of variances, and Bonferroni’s post hoc test was utilized to ascertain differences between identified clades. Regression analyses for time dependence, as well as dependence on yearly sunshine hours, yearly precipitation and monthly average temperatures in central Cyprus (Mesaoria Plain, the vicinity of Nicosia), were conducted using the quadratic and cubic models, and the better-fitting model was selected.

#### Exclusions

The phytochemicals marked with a dagger in Table 2 and Table 3 were excluded from PCA and ANOVA, as they were detected only in the mature leaves and not the juveniles; therefore, PCA and ANOVA could not be conducted on them.

## 4. Conclusions

The present study is among the pioneers of its kind in the genus *Eucalyptus*, compounding the use of Hierarchical Cluster Analysis, determining the primary components of variability and conducting statistical analysis on *E. torquata*. The temporal variation on the chemical composition of the essential oil of *E. torquata* is also reported herein for the first time. The essential oil yields peaked approximately at 3.2%, which was relatively higher than the previous literature. The samples for the essential oils of both leaf types for all months were dominated by 1,8-cineole (in mature leaves: 3.6–27.8%; in young leaves: 12.7–21.5%) and torquatone (in mature leaves: 27.6–48.8%; in young leaves: 28.8–41.5%) which were the major compounds, and there was a negative relationship between the content torquatone and 1,8-cineole and α-eudesmol, β-eudesmol and α-pinene (*p* < 0.001, *p* = 0.021, *p* = 0.012 and *p* = 0.007, respectively). The results unveiled the existence of three major clusters, which can shed light on the chemical evolution of the essential oil of *E. torquata* over the span of a year. The presence of these clusters indicates that *E. torquata* passes through certain stages of development, with pulses of miniatone production in late winter and early spring in the juvenile leaves, associated with Cluster B. The miniatone spike was not observed with mature leaves, which did not present with any members in the miniatone-enriched Cluster B.

## Data Availability

Data are contained within the article and Supplementary Materials.

## Supplementary Materials

The following supporting information can be downloaded at: https://www.mdpi.com/article/10.3390/molecules30020332/s1, Rotated Component Matrix.
